# Study on the degradation pathway of benzo[a]pyrene by *Bacillus* sp. strain M1

**DOI:** 10.3389/fmicb.2025.1633648

**Published:** 2025-09-08

**Authors:** Xiaoxue Zhang, Guohui Ning, Mingyue Qi, Jiahui Li, Xuena Zhang, Rui Hao, Xuefeng Zhao, Xiaomin Wang, Zhixin Yang

**Affiliations:** ^1^State Key Laboratory of North China Crop Improvement and Regulation, Ministry of Education of China-Hebei Province Joint Innovation Center for Efficient Green Vegetable Industry, Hebei Agricultural University, Baoding, China; ^2^Center for Wetland Conservation and Research, Hengshui University, Hengshui, China; ^3^Ordos Wulan Coal Group Co., Ltd., Ordos, China; ^4^College of Resource and Environmental Sciences and Key Laboratory for Farmland Eco-Environment, Agricultural University of Hebei, Baoding, China

**Keywords:** *Bacillus*, benzo[a]pyrene degradation, metabolism, key enzymes, phthalic acid pathway

## Abstract

*Bacillus* sp. strain M1, originally isolated from soil contaminated with polyaromatic hydrocarbons during earlier work of our group, has a strong ability to degrade benzo[a]pyrene (BaP). Here we show that the bacteria can utilize phthalic acid, protocatechuic acid, salicylic acid, catechol or gentisic acid as the sole carbon source, with utilization of 65.50–92.28% within 2 days. The metabolites formed during culture of *Bacillus* sp. strain M1 in mineral salt medium with BaP as the sole carbon source were detected by GC–MS. Intermediate metabolites were identified that included 4,5-dimethylphenanthrene, 9-ethyl-10-methylanthracene, 2-ethylacridine, phthalate derivatives, silylated catechol, and salicylic acid. The involvement of six relevant intracellular enzymes, namely phthalate dioxygenase, salicylate hydroxylase, catechol-1,2-dioxygenase, catechol-2,3-dioxygenase, gentisic acid dioxygenase and dehydrogenases, was demonstrated, and their activities correlated with the level of BaP degradation. Incorporating all findings, we propose a degradation pathway of BaP by strain M1 that starts with an enzymic attack of the C-9/C-10 or the C-7/C-8 bond. The pathway is summarized as BaP → cis-4,5-pyrene dihydrodiol → 4,5-dimethyl phenanthrene → 1-hydroxy-2-naphthalic acid. In addition, two new products, 9-ethyl-10-methylanthracene and 2-ethylacridine, were discovered.

## 1 Introduction

Benzo[a]pyrene (BaP) is a high-molecular weight polycyclic aromatic hydrocarbon (HMW-PAH) containing five aromatic rings. It demonstrates low aqueous solubility, high lipophilicity, and environmental persistence (Rodríguez-Fragoso et al., 2009). It has carcinogenic, immunotoxic and mutagenic properties, and upon entering human cells, it is activated by multifunctional oxidases in cellular microsomes. The World Health Organization (WHO) has categorized BaP as a primary carcinogen (World Health Organization, 2012).

Certain microorganisms can degrade PAHs in the environment to form less toxic products through a series of enzyme-catalyzed reactions (Seo et al., 2009). Microbial degradation pathways have been predicted by identifying the produced intermediate metabolites during PAH degradation and by demonstrating induced expression of key enzymes involved in the process (Selvakumaran et al., 2011; Sun et al., 2019). Most of the research carried out on microbial metabolic pathways of PAH degradation has focused on low molecular weight PAHs, such as naphthalene and phenanthrene, and their degradation pathways are well characterized (Wang et al., 2018). Three main pathways for naphthalene degradation by microorganisms have been identified, namely via meta-catechol cleavage, ortho-catechol cleavage and the gentisic acid pathway (Peng et al., 2008; Seo et al., 2009). All three pathways start with naphthalene dioxygenase, an enzyme that uses naphthalene to form 1,2-dihydroxynaphthalene, after which the naphthalene ring is opened by a series of reactions to produce salicylic acid. After this metabolite is converted to catechol or to gentisic acid by hydroxylases, these are further degraded to eventually enter the tricarboxylic acid (TCA) cycle. Phenanthrene (PHE) can be aerobically degraded via two main pathways, in which salicylic acid (via the meta-cleavage pathway) or phthalic acid (via the ortho-cleavage pathway) is produced, respectively (Prabhu and Phale, 2003; Seo et al., 2009; Waigi et al., 2015). First, phenanthrene dioxygenase catalyzes the oxygen attack at positions 3,4 of PHE, to form cis-3,4-phenanthrene dihydrodiol, or, alternatively, an attack on the 1,2 positions of PHE forms cis-1,2-phenanthrene dihydrodiol. These intermediates are used by dihydrodiol dehydrogenase to form 3,4-dihydroxyphenanthrene and 1,2-dihydroxyphenanthrene, respectively. These are then are further degraded to salicylic acid (in the meta-cleavage pathway) or phthalic acid (in the ortho-cleavage pathway). Salicylic acid mineralization involves the activity of salicylate hydroxylase and salicylate-5-hydroxylase. For mineralization of phthalic acid, as many as 7 different enzymes have been identified: phthalate dioxygenase, 3,4-dihydroxyphthalate dehydrogenase, 3,4-dihydroxyphthalate decarboxylase, 4,5-dihydroxyphthalate dehydrogenase, 4,5-dihydroxyphthalate decarboxylase, 3-carboxy-cis,cis-muconate oxygenase, and 4-carboxy-2-hydroxymuconate semialdehyde oxygenase. Both pathways eventually allow the breakdown products to enter the TCA cycle through catechol (Prabhu and Phale, 2003; Waigi et al., 2015) so that PHE is completely mineralized into carbon dioxide and water, while providing energy for the bacteria Bacterial stains do not necessarily employ one degradation pathway only, as some may operate two or more phenanthrene metabolic pathways at the same time (Seo et al., 2012).

Due to its relative stability, BaP is not easily degraded by microbes, and few metabolic degradation pathways have been recognized. In case bacterial degradation has been demonstrated, intermediate metabolites have been identified, including cis-4-7(−hydroxypyridine-8-yl)-2-oxobut-3-enoic acid, cis-4,5-BaP-dihydrodiol, cis-7,8-BaP-dihydrodiol, 4-methylphenanthrene, 2,3-dimethylphenanthrene, and diethyl phthalate (Chen et al., 2013; Liang et al., 2014; Mohandass et al., 2012; Qin et al., 2018; Qin et al., 2017; Zang et al., 2006). Based on these and other identified metabolites, three metabolic pathways have been proposed that would degrade 5-ring BaP into 4-ring pyrene (Pyr) derivatives. The first pathway generates cis-7,8-dihydro-dihydroxy-BaP under the action of a dioxygenase as a first step, and after re-aromatizing this into cis-7,8-dihydroxy-BaP by a dehydrogenase, it is further opened by a dioxygenase to generate 7-hydroxy-8-carboxy-Pyr. This intermediate then enters the Pyr degradation pathway. The second way to degrade BaP is highly similar, but proceeds via cis-9,10-dihydro-dihydroxy-BaP and cis-9,10-dihydroxy-BaP, which is then opened to generate 9-hydroxy-10-carboxy-Pyr, before it is further degraded. Similarly, the third pathway proceeds via cis-4,5-dihydro-dihydroxy-BaP, cis-4,5-dihydroxy-BaP and 4,5-chrysenedicarboxylic acid, which continues to be degraded.

When pyrene derivatives are formed, they are further degraded by dioxygenation at the 4,5-positions, to produce both cis- and trans-4,5-pyrenedihydrodiol, by a dioxygenase and a monooxygense, respectively. Rearomatization of the dihydrodiol and subsequent ortho-cleavage then leads to the formation of 4,5-dicarboxyphenanthrene, which is further decarboxylated to give 4-phenanthroate. Following another di-oxygenation reaction, 4-phenanthroate forms cis-3,4-dihy- droxyphenanthrene-4-carboxylate. Rearomatization of this metabolite yields 3,4-dihydroxyphenanthrene, which is further metabolized to 1-hydroxy-2-naphthoate. The subsequent enzymatic reactions, including intradiol ring cleavage dioxygenation, result in the production of phthalate. This is further transformed to enter the TCA cycle via the β-ketoadipate pathway (Li et al., 2022). The microorganisms that have been identified to employ this metabolism of BaP include *Mycobacterium* sp. *RJGII-135*, *Beijerinckia* sp., *Mycobacterium, Rhodococcus*, and others.

These proposed degradation pathways of BaP are based on limited evidence of identified intermediate metabolites (Chen et al., 2013; Liang et al., 2014; Peng et al., 2012; Qin et al., 2018; Qin et al., 2017). However, due to their rapid turnover, intermediate metabolites do not accumulate and can be difficult to separate in sufficient quantities required for identification. As a result, the actual metabolic steps of BaP breakdown by a given bacterial strain have been precisely defined. For this, it is necessary to identify more intermediate metabolites produced during BaP degradation. Moreover, the pathway(s) of BaP degradation can vary between different species and strains. Some work has been carried out with *Pseudomonas* and *Microbacterium* strains, or with the species *Cellulosimicrobium cellulans* and *Stenotrophomonas maltophilia* (Chen et al., 2013; Liang et al., 2014; Qin et al., 2018; Qin et al., 2017). Other bacterial species and strains that are able of BaP metabolism still need to be investigated, in order to evaluate the complete array of pathways that can lead to biological PAH degradation.

Our research group has identified a *Bacillus* sp. isolate designated strain M1, that was originally isolated from agricultural soil contaminated by coal mining practices (Wang, 2016; Zhang, 2017), with a high efficiency for HMW-PAHs degradation. The removal rate of BaP by this strain was 54% after 7 days of growth in medium containing 10 mg L^−1^ BaP as the sole carbon source, and as high as 90% in rich culture medium with BaP. However, the intermediate metabolites produced or the enzymes involved in this metabolism had not yet been identified. To fill this knowledge gap, here we present data on intermediate metabolites detected by gas chromatography–mass spectrometry (GC–MS) analysis. Specifically, strain M1 was grown on media containing defined organic acids as the sole carbon source (salicylic acid, phthalic acid, protocatechuic acid, gentisic acid or catechol), in order to identify whether the salicylic acid or the phthalic acid pathway was involved. The intermediate metabolites of M1 degrading BaP and the key enzyme activities involved were then identified. An attempt was made to construct the metabolic chain of events that leads to degradation of BaP by *Bacillus* sp. strain M1, thereby providing a theoretical basis for a novel HMW-PAH degradation pathway.

## 2 Materials and methods

### 2.1 Chemicals, media and bacteria used

BaP was ≥99% purity (AccuStandard, Inc., United States). A stock solution in acetone (1 g L^−1^) was prepared and stored at −20 °C. N-hexane, acetone, ethyl acetate, *p*-terphenyl-d14 standard, 4-bromo-2-fluorobiphenyl salicylic acid, phthalic acid, 1-hydroxyl-2-naphthalic acid, and 2,5-dihydroxybenzoic acid were obtained from J&K Scientific and Sinopharm Chemical Reagent Co., Ltd., Beijing. All other chemicals were obtained at the highest purity grade available from ANPEL Laboratory Technologies (Shanghai).

Mineral salt medium (MSM) containing 5.0 mg L^−1^ BaP was prepared as previously described (Zhang et al., 2022). Organic acids were added as indicated and the pH of the medium was adjusted to 7.5. Starter cultures of *Bacillus* sp. M1 were grown in Luria-Bertani medium (LB, pH 7.5) containing 10 g peptone, 5 g yeast extract, 10 g NaCl and 1 L distilled water.

*Bacillus* sp. strain M1 was originally isolated from agricultural soil derived from a coal mining area and was characterized as previously described (Zhang, 2017). Starter cultures were produced from a frozen stock in 100 mL LB medium, incubated in a rotary shaker at 150 rpm, 30 °C for 24 h. After centrifugation (5,000 rpm, 15 min) and 2 to 3 washes with sterile MSM, the bacteria were resuspended in sterile distilled water at an OD_600_ of 1.0, equivalent to 3.0 × 10^9^ cells mL^−1^ as determined by plate counts, to be used as the inoculum for the experiments.

### 2.2 Experimental setup

#### 2.2.1 Culturing strain M1 with low-molecular weight organic acids

A total of 5 low molecular weight organic acids, namely salicylic acid, phthalic acid, protocatechuic acid, gentisic acid, and catechol, were tested to assess their utilization as the sole carbon source by strain M1 during growth. For this, 5 mL of M1 suspension (prepared as described above) was added to a 250 mL flask containing 100 mL MSM with 100 mg L^−1^ of each of the LMW acid as the sole carbon source. A non-inoculated control was included for each treatment and experiments were performed in triplicate. All treatments were incubated in the dark at 30 °C on a rotary shaker at 150 rpm. Samples were collected at days 0, 1, 2, 3 and 4. Following centrifugation at 5,000 rpm for 15 min, the residual contents of the carbon source in the culture supernatant was determined as described below (section 2.3.1).

#### 2.2.2 Culturing strain M1 with BaP

Cultures were set up to assess extracellular intermediate metabolites and enzyme activities in presence and absence of BaP during cultivation. For this, 1 mL of bacterial suspension was added to 20 mL MSM in a 100 mL Erlenmeyer flask and BaP was added to a final concentration of 5 mg L^−1^. A blank control was included without BaP but with M1 strain inoculation, and another control contained BaP without bacteria. Each treatment was performed with six replicates. All samples were incubated at 30 °C on a rotary shaker at 150 rpm for 7d.

Every day for 7 days, a sample was collected from three replicates to determine the presence of BaP (section 2.3.2) and of extracellular BaP intermediates (section 2.3.3). Simultaneously, the other three replicates were used to prepare a crude cell extract as described in section 2.4.

### 2.3 Analysis of organic compounds

#### 2.3.1 Utilization of low-molecular weight organic acids

The supernatants obtained as described in section 2.2.1 were centrifuged at 4 °C, 11,000 rpm for 10 min and the resulting supernatant was used for the determination of residual contents of salicylic acid, phthalic acid, gentisic acid, protocatechuic acid and catechol by ultraviolet spectrophotometry at 296 nm, 292 nm, 320 nm, 320 nm, and 275 nm, respectively (Bosch et al., 1999; Rha et al., 2019; Yan and Ye, 1995).

#### 2.3.2 Utilization of BaP

The amount of BaP remaining in the culture supernatants obtained from the experiments described in 2.2.2 was determined by GC–MS (Aglient 7890/5975c) as described in the literature (Zhang et al., 2020). Briefly, extraction of BaP from the supernatants was done using n-hexane/acetone with two internal standards present. The oven temperature started at 80 °C for 2 min, to then linearly increase with 5 °C min^−1^ to 140 °C; after 3 min it was further increased to 210 °C, and after another 3 min, the temperature was increased with 5 °C min^−1^ to 290 °C and kept constant for 3 min. The inlet temperature was 280 °C, the ion source temperature was 230 °C, and the quadrupole temperature was 150 °C (Zhang et al., 2020).

Calibration curves were produced with 1, 2, 5, 10 and 20 μg L^−1^ BaP to calculate the BaP concentrations of the extracts by the relative ratios of peak areas.

Quality assurance protocols and the recovery percentage requirements were performed as described in the literature (Zhang et al., 2020).

#### 2.3.3 Determination of BaP metabolites

The supernatants obtained as described in section 2.2.2 were used to extract intermediate metabolites of BaP. The pH of the supernatants was adjusted to 1.2 to 2 with 6 mol/L HCl. Then ethyl acetate was added, mixed and shaken at 150 rpm for 20 min. After separation of the phases, the upper organic phase was collected and ethyl acetate extraction was repeated with the aqueous lower phase, after which both organic phases were combined. This extract was dried with anhydrous Na_2_SO_4_, followed by nitrogen gas at 35 °C. After dissolving the solids in 1 mL acetone, the extracts entered a 2 mL headspace sample bottle for testing. The intermediate metabolites of BaP were analyzed by GC–MS with full-spectrum scanning (Tao et al., 2007). The chromatographic conditions were as follows: the ion source was 230 °C and the quadrupole temperature was 150 °C; the initial temperature was kept at 50 °C for 2 min, to then linearly increase with10 °C min^−1^ to 300 °C and kept constant for 6 min. The mass spectrum was scanned for a range of 50 ~ 1,000 (Chen, 2014; Moscoso et al., 2012).

### 2.4 Enzyme activity analyses

For enzyme activity determination, bacterial cells were lysed by sonication on ice with 400 W for 3 s, with intermittent cooling of 3 s for a total of 15 min, as previously described (Zhang et al., 2020). After centrifugation, the supernatant was used to determine enzyme activity of catechol-1,2-dioxygenase, catechol-2,3-dioxygenase, alicylate hydroxylase, phthalate dioxygenase, gentisate 1,2-dioxygenase and dehydrogenase. In the first 5 cases, 3 mL reaction solutions (described below) were prepared in phosphate buffer (pH 7.5) and UV-spectrophotometry was used to determine enzymic activity in presence of the required substrate. The activity of dehydrogenases was measured using the 2,3,5,triphenyl tetrazolium chloride (TTC) method. In all cases, except for salicylate hydroxylase, 1 unit of enzyme activity was defined as the amount of enzyme required to generate 1 μmol min^−1^ of product at the indicated temperature.

Activity of catechol-1,2-dioxygenase (EC 1.13.11.1) was measured after the addition of 100 μmol L^−1^ catechol. During incubation at 35 °C, over time 1 mL aliquots were taken and used to measure the amount of cis,cis-muconic acid, the oxidation product of catechol produced by this enzyme. This has an extinction coefficient ɛ_260nm_ of 16,800 L mol^−1^ cm^−1^ at 260 nm (Guzik et al., 2013).

The activity of catechol-2,3-dioxygenase (EC 1.13.11.2) was determined similarly, but the incubation was performed at 30 °C and the time interval of sampling was 30s. The product, 2-hydroxymuconic semialdehyde, was quantified at 375 nm with ɛ_375nm_ = 36,000 L mol^−1^ cm^−1^ (Wojcieszyńska et al., 2012).

Activity of salicylate hydroxylase (EC 1.14.13.1) was measured in presence of 100 μmol L^−1^ salicylic acid and 100 μmol L^−1^ NADH. Consumption of NADH was monitored at 340 nm with ɛ_340 nm_ = 6,220 L mol^−1^ cm^−1^, and activity units were defined as the amount of enzyme required to consume 1 μmol NADH per minute at 30 °C (Zhou et al., 2002).

Phthalate dioxygenase (EC 1.14.12.7) was determined by adding 100 μmol L^−1^ phthalic acid as a substrate in presence of NADH. Production of the enzymic product cis-4,5-dihydroxycyclohhexa-1(6),2-diene-1,2-dicarboxylate was monitored at 275 nm (ɛ_275 nm_ = 14,700 L mol^−1^ cm ^−1^) (Zeinali et al., 2008).

Gentisate 1,2- dioxygenase (EC1.13.11.4) was measured with 100 μmol L^−1^ gentisic acid as a substrate, from which maleylpyruvate was produced that was monitored at 330 nm (ɛ_330 nm_ = 10,800 L mol^−1^ cm^−1^) (Pumphrey and Madsen, 2007).

Dehydrogenase activity was demonstrated by the TTC method. For this, the bacterial culture was added to 2 mL 0.1 mol L^−1^ Tris–HCl (pH 8.5) containing 0.1 mol L^−1^ glucose and 0.5% TTC and incubated for 24 h at 30 °C. The reaction was terminated by adding 2 drops of H_2_SO_4_ and the formed product was extracted with toluene, to be measured as absorbance at 486 nm as described elsewhere (Gao et al., 2016; Xiao et al., 2019).

### 2.5 Statistical analysis

The utilization efficiency U_s_ of substrate was calculated as *U_s_* = (*C*_0_–*C_t_*)/C_0_ × 100%, where *C*_0_ is the initial concentration of a substrate and *C*_t_ is the residual concentration at the time of sampling. Likewise, the removal efficiency *R_s_* of BaP was calculated as *R_s_* = (*C*_0_–*C_t_*)/C_0_ × 100%.

Statistical analysis was performed using Excel, SPSS 17.0. The differences among the treatments were analyzed by one-way ANOVA (LSD test).

## 3 Results

### 3.1 Utilization of added organic acids by strain M1

Salicylic acid, gentisic acid and catechol are intermediate metabolites in the biological degradation of PAHs through the salicylic pathway, while phthalic acid and protocatechuic acid are intermediates of the phthalic acid pathway. To investigate the involvement either of these pathways during BaP degradation by *Bacillus* sp. strain M1 it was first tested whether the bacteria could utilize these compounds as the sole carbon source.

**Figure 1:**
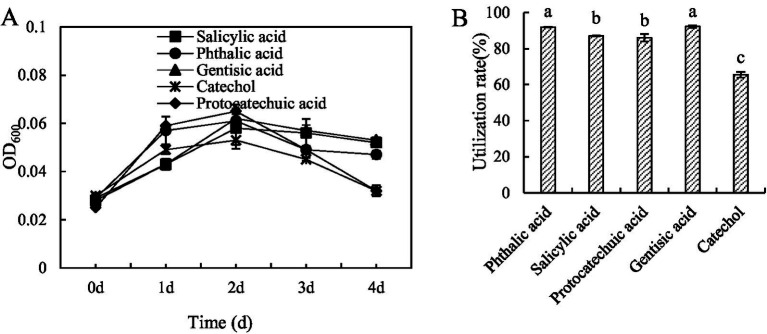
Growth curves **(A)** and utilization **(B)** of *Bacillus* sp. strain M1 cultured in medium with 5 different low molecular weight organic acids as the sole carbon source. Statistical significance between different treatments, is indicated in panel **(B)** with non-identical letters.

**Figure 2:**
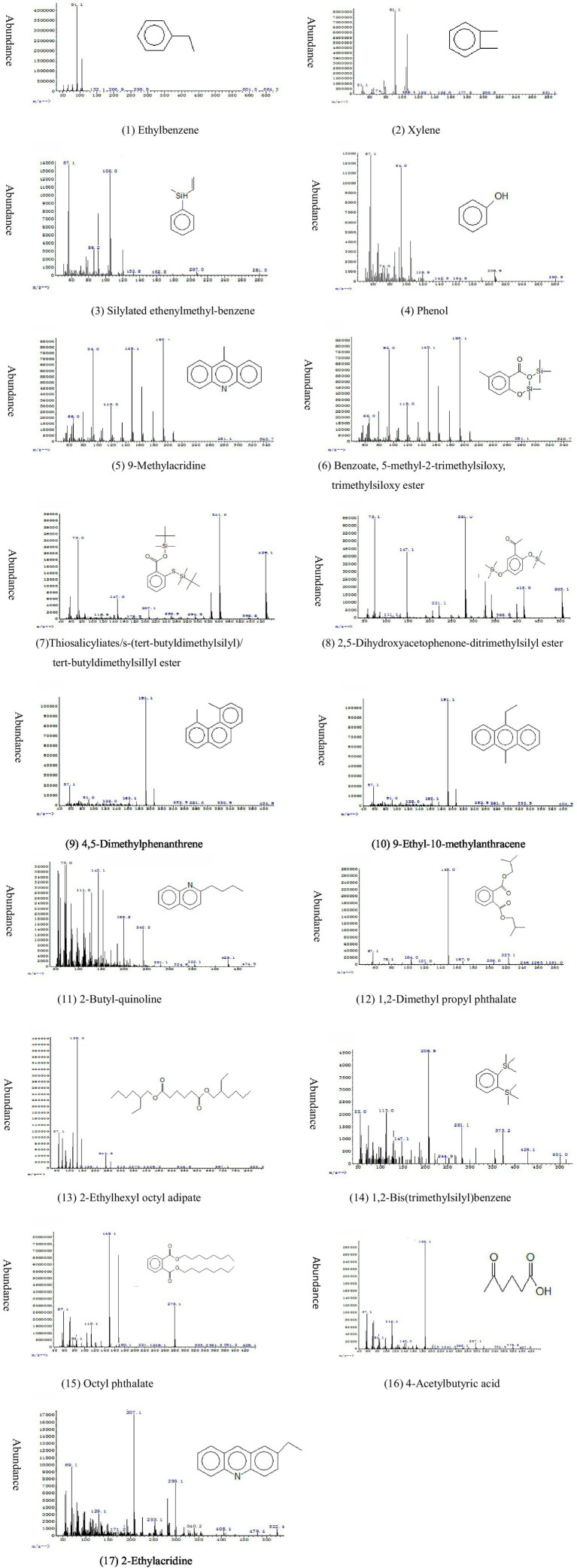
Mass spectra and structures of 16 identified compounds in cultures of *Bacillus* sp. strain M1 grown in presence of BaP as the sole carbon source. All spectra are shown after correction for the blank. For explanation, see text.

Strain M1 was able to grow on all 5 substrates, with growth curves shown in Figure 1. Highest cell densities, as indicated by OD_600_ measurements, were obtained on day 2, after which cell density decreased (Figure 1A). With protocatechuic acid and gentisic acid as a carbon source, growth at day 1 was significantly higher than that with the other three substrates, while growth on phthalic acid and gentisic acid was weakest. The amount of carbon substrate utilized at day 2 was 91.88% for phthalic acid and 92.28% for gentisic acid, slightly (but statistically significantly) lower for salicylic acid and protocatechuic acid (87.08 and 86.02% respectively) and lower still, with statistical significance, for catechol (65.50%) (Figure 2B). This indicated that phthalic acid and gentisic acid are easier to utilize by strain M1 than the other three tested carbon sources. According to these results, it is possible that BaP degradation by strain M1 involves either the salicylic acid pathway or the phthalic acid pathway, or even that both pathways might co-exist simultaneously.

### 3.2 Intermediate metabolites produced during degradation of BaP by strain M1

When strain M1 was cultivated in culture medium containing BaP as the sole carbon source for 7 days, the BaP content decreased from 5.86 μg L^−1^ on day 0 to 0.75 μg L^−1^ on day 7 (Table 1), indicating that BaP was degraded over time.

**Table 1:** Response peaks and concentration changes of BaP over time during culture of *Bacillus* sp. strain M1.

Culture time (d)	Control (BaP)		Treatment(BaP + M1)	
	BaP (μg/mL)	Response peak area	BaP + M1(μg/mL)	Response peak area
0	5.71 ± 0.17	6,341,157 ± 47,326	5.86 ± 0.13	6,526,693 ± 56,214
1	4.79 ± 0.35	5,397,611 ± 91,499	1.92 ± 0.20	1,896,260 ± 202,850
2	4.10 ± 0.24	3,894,885 ± 53,548	1.28 ± 0.13	973,742 ± 167,517
3	4.81 ± 0.10	4,116,004 ± 96,792	1.39 ± 0.12	957,034 ± 125,163
4	4.73 ± 0.09	3,780,357 ± 74,056	1.17 ± 0.08	703,148 ± 86,981
5	4.04 ± 0.23	4,375,344 ± 226,563	1.10 ± 0.04	878,168 ± 10,771
6	4.40 ± 0.06	4,957,528 ± 77,197	0.81 ± 0.00	554,294 ± 3,523
7	4.30 ± 0.32	4,876,267 ± 76,340	0.75 ± 0.04	485,104 ± 7,835

GC/MS was used to determine extracellular intermediate metabolites of BaP. The chromatographic peaks in the produced chromatograms were analyzed and identified according to the peak characteristics of ion fragments in mass spectrometry. The structure of ion fragments were compared with NIST08. L data and published literature for identification. The peak area and fragment ion characteristics are summarized in Table 2 and the spectra with the most likely deduced molecular structure is summarized in Figure 2. The intermediate metabolites detected on day 1 of the culture include 1,2-bis(trimethylsilyl)benzene, 2-ethylacridine, thiosalicylic acid / s-(tert-butyldimethylsilyl)/tert-butyldimethylsillyl ester, and 2,5-dihydroxyacetophenone-ditrimethylsilyl ester (numbers 14, 17, 7, and 8 in Table 2 and Figure 2). On day 2, 4,5-dimethylphenanthrene (number 9) and 9-methylacridine (number 5) were detected, while on day 3 9-ethyl-10-methylanthracene (number 10) and on day 6 4-acetylbutyric acid (number 16) were detected. Compounds detected on every day of the culture included ethylbenzene, xylene, silyated ethenylmethyl-benzene, phenol, 2-butyl-quinoline, 1,2-dimethyl propyl phthalate, 2-ethylhexyl octyl adipate and octyl phthalate (numbers 1, 2, 3, 4, 11, 12, 13 and 15), and the accumulated amounts of these substances in the medium first increased and then decreased over time. Thus, strain M1 produced various detectable extra cellular metabolites when growing in BaP-containing medium. Notably, some metabolites were transient, detectable only at specific time points only, while others appeared at different stages of the incubation and remained present for some time. This could be due to different turnover rates of the metabolites, low amounts of accumulation or concentrations below detection levels.

**Table 2:** The main intermediate products detected in the degradation process of BaP.

Number	Peak area	Fragment ion characteristics	Possible structure	Days it was detectable (structure before silanization)
1	106,403,606	91.1, 106, 77	Ethylbenzene/Xylene	Day 1–7
2	260,584,588	91.1, 106, 98	Ethylbenzene/Xylene	Day 1–7
3	1,156,057	105, 120, 86.2	Silylated ethenylmethyl-benzene	Day 1–7 (ethenylmethyl-benzene)
4	26,971,609	94, 66, 119.9	Phenol	Day 1–7
5	1,294,933	193.1, 149.1, 94	9-Methylacridine	Day 2
6	2,195,456	281, 94, 149	Benzoate/5-methyl-2-trimethylsiloxy/trimethylsiloxy ester	2-hydroxy-5-methylbenzoic acid
7	475,613	341, 429, 73	Thiosalicylic acids/s-(tert-butyldimethylsilyl)/tert-butyldimethylsillyl ester	Day 1 (salicylic acid)
8	1,024,217	281, 73, 147	2,5-Dihydroxyacetophenone-ditrimethylsilyl ester	Day 1 (2,5-dihydroxyacetophenone)
9	2,035,220	191.1, 206, 175	4,5-Dimethylphenanthrene	Day 2
10	405,636	205.2, 220, 191	9-Ethyl-10-methylanthracene	Day 3
11	1,734,531	143.1, 73, 154.1	2-Butyl-quinoline	Day 1–7
12	4,579,685	149, 57.1, 223.1	1,2-Dimethyl propyl phthalate	Day 1–7
13	17,443,030	129, 57.1, 241.2	2-Ethylhexyl octyl adipate	Day 1–7
14	3,265,562	206.9, 113, 281	1,2-Bis(trimethylsilyl)benzene	Day 1 (catechol)
15	695,976,450	149.1, 167, 279	Octyl phthalate	Day 1–7
16	167,832	185.1, 112, 203	4-Acetylbutyric acid	Day 6
17	375,524	299.2, 207, 129	2-Ethylacridine	Day 1

Of the identified intermediates, 4,5-dimethylphenanthrene (number 9), 9-ethyl-10-methylanthracene (10), 2-butyl-quinoline (11), 2-ethylacridine (17) and 9-methylacridine (5) contain 2 to 3 benzene rings, which were likely formed from HMW-BaP. A number of compounds were derived by silylation during GC/MS detection, such as silyated ethenylmethyl-benzene (number 3), 2,5-dihydroxyacetophenone-ditrimethylsilyl ester (8), benzoate, 5-methyl-2-trimethylsiloxy, trimethylsiloxy ester (6), thiosalicyliate, s-(tert-butyldimethylsilyl), tert-butyldimethylsilyl ester (7), and 1,2-bis(trimethylsilyl)benzene (14). These contained at least one trimethylsilyl group (Si(CH_3_)), which was produced by replacing a hydrogen atom of a carboxyl or phenolic hydroxyl group. Thus, their presence indicated that the possible corresponding substances before substitution were ethenylmethyl-benzene (for 3), 2,5-dihydroxyacetophenone (for 8), 2-hydroxy-5-methylbenzoic acid (for 6), salicylic acid (for 7) and catechol (for 14). These findings were consistent with the finding that M1 could utilize both salicylic acid and catechol. In addition, we detected heterocyclic compounds containing sulfur and nitrogen (numbers 5, 7, 11, 14 and 17, see Figure 2), compounds that were likely derived from the MSM medium. None of the above 17 intermediate metabolites were detectable in the blank control that had not contained strain M1.

Esters containing a phthalate group (numbers 6, 7, 8, 12, 13 and 15) were detected in different forms in the supernatants from day 1 to 7. These may have been the result of fragmentation and rearrangement of intermediate metabolites during mass spectrometry. The mass spectrum response intensity, as well as the chromatographic peak area, of phthalic acid esters was significantly higher than that of the other substances. This would suggest these substances accumulated at relatively large quantities during degradation of BaP, and they would represent the main metabolites of BaP degradation by strain M1. This conclusion is consistent with the finding that M1 can use phthalic acid and catechol as a carbon source, and these findings corroborate the interpretation that the degradation of BaP by M1 can be accomplished through the phthalic acid pathway. In view of the high phthalate peaks, we consider the phthalic acid pathway to be the main, but not necessarily the sole pathway for strain M1 to metabolize BaP.

In summary, *Bacillus* sp. strain M1 can metabolize BaP through both the salicylic acid pathway and the phthalic acid pathways, with the latter probably functioning as the main metabolic pathway. Although metabolites containing 2–3 benzene rings were detected, no aromatic metabolites with 4 benzene rings were identified.

### 3.3 Enzyme activities and the removal of BaP during metabolism

**Figure 3:**
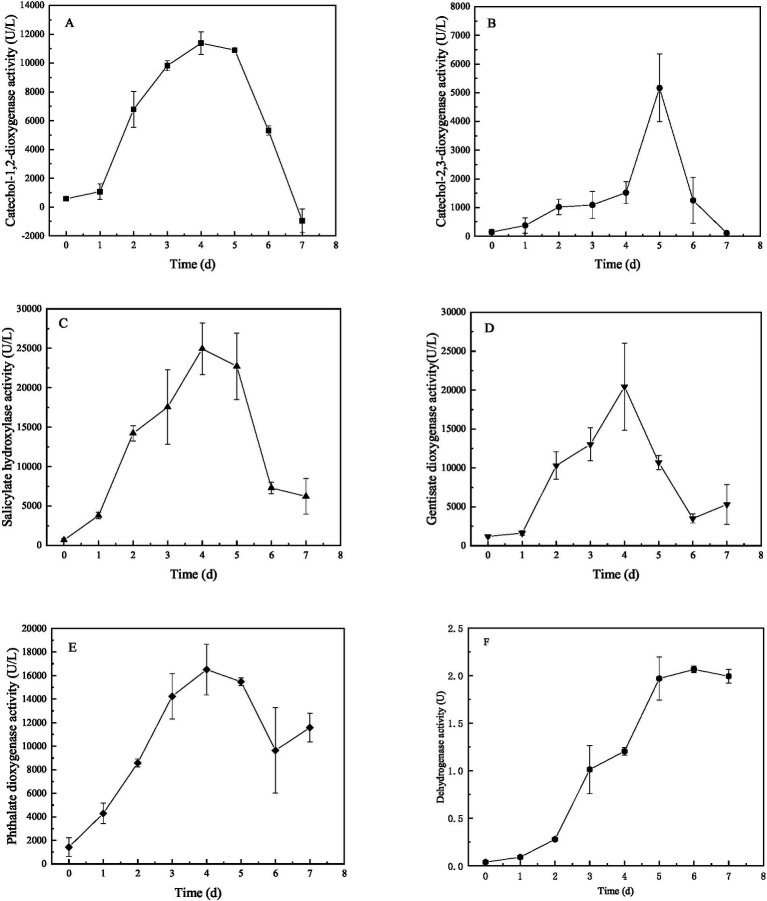
Enzyme activity over time during cultures of *Bacillus* sp. strain M1 in presence of BaP as the sole carbon source, for catechol-1,2-dioxygenase **(A)**, catechol-2,3-dioxygenase **(B)**, salicylate hydroxylase **(C)**, gentisate dioxygenase **(D)**, phthalate dioxygenase **(E)**, and dehydrogenases **(F)**.

The crude cellular extracts obtained from M1 cultures in presence of BaP were assessed for enzyme activities *in vitro*. Enzyme activity could be demonstrated for phthalate dioxygenase, salicylate hydroxylase, gentisate dioxygenase, catechol-1,2-dioxygenase, catechol-2,3-dioxygenase and non-specified dehydrogenases. Their activity changed with time of culturing (Figure 3). All activities increased during the first 4 days of culturing, to variable degrees, after which enzyme activity decreased, with the exception of dehydrogenases (Figure 3F). The determined activities of phthalate dioxygenase, salicylate hydroxylase and catechol-1,2-dioxygenase activity was high during days 4 ~ 5 and was significantly higher than the enzyme activity at other days, suggesting enzyme activity was regulated. The activity of phthalate dioxygenase (Figure 3E) reached 16,508 U L^−1^ at day 4, which was 11.49-folds higher than the activity at day 0. Gentisate dioxygenase activity (Figure 3D) also varied significantly over time, reaching a peak at day 4 of 20,453 U/L, which was 17.14-folds higher than the activity at day 0. Salicylate hydroxylase (Figure 3C) and catechol-1,2-dioxygenase activities peaked at 24,938 U/L and 11,376 U/L respectively, which was 36.74- and 19.55-folds higher than that of at day 0, respectively. Lastly, dehydrogenase activity (Figure 3F) continuously increased after day 1 to reach a plateau at day 5. There were significant differences among the five oxidases at the same incubation day. Phthalate dioxygenase activity was significantly higher than that of the other four enzymes and catechol 2, 3-dioxygenase activity was the lowest (*p* < 0.05).

Based on the consistency between the activities of the six enzymes and the removal of BaP from the culture, fitting models were further applied that confirmed a correlation between BaP degradation and the enzyme activity, resulting in significant linear positive relationships with high confidence levels (Table 3). This supports the hypothesis that the six enzymes produced by *Bacillus* sp. M1 were performing key function in the degradation of BaP. The highest correlation was observed for salicylate hydroxylase (*R* = 0.9131) and phthalate dioxygenase (*R* = 0.8422), while the lowest correlation was found for catechol-2,3-dioxygenase (*R* = 0.5698). Thus, although the latter is a key enzyme, its activity does not correlate strongly with concentrations of BaP, possibly because it stays active when BaP concentrations are already decreasing, or because it has other functions in the cell as well. These results also indicated that these enzymes participated in different stages of M1 metabolizing BaP, causing a gradual BaP oxidation. Among the six enzymes, phthalate dioxygenase has the strongest enzymatic activity and it produced a strong correlation, so it probably played a leading role in BaP degradation by M1 through the phthalate pathway. At the same time, enzymes involved in the salicylic acid pathway are also actively involved; for instance, BaP concentrations strongly correlated with salicylate hydroxylase, which is consistent with the results of the intermediate metabolites produced by M1 to degrade BaP.

**Table 3:** Correlation analysis between BaP residual content and five enzyme activities during culturing of strain M1.

Enzyme activity	Binary linear equation^a^	*R*
Phthalate dioxygenase	y = 3E-08x^2^–0.0007x + 5.6895	0.8422***
Salicylate hydroxylase	y = 62.269x^−0.399^	0.9131***
Gentisate dioxygenase	y = 55.21x^−0.394^	0.7645***
Catechol-2,3-dioxygenase	y = 9E-07x^2^–0.0031x + 3.8934	0.5698*
Catechol-1,2-dioxygenase	y = 29.751x^−0.343^	0.7643***
Dehydrogenase	y = 1.1423x^−0.358^	0.8014***

a y is the BaP residual content (ug/mL); x is the enzyme activity.

* indicates a significance level of 5%;** indicates a significance level of 1%;*** indicates a significance level of 0.1%.

### 3.4 A proposed metabolic pathway of BaP degradation by M1

**Figure 4:**
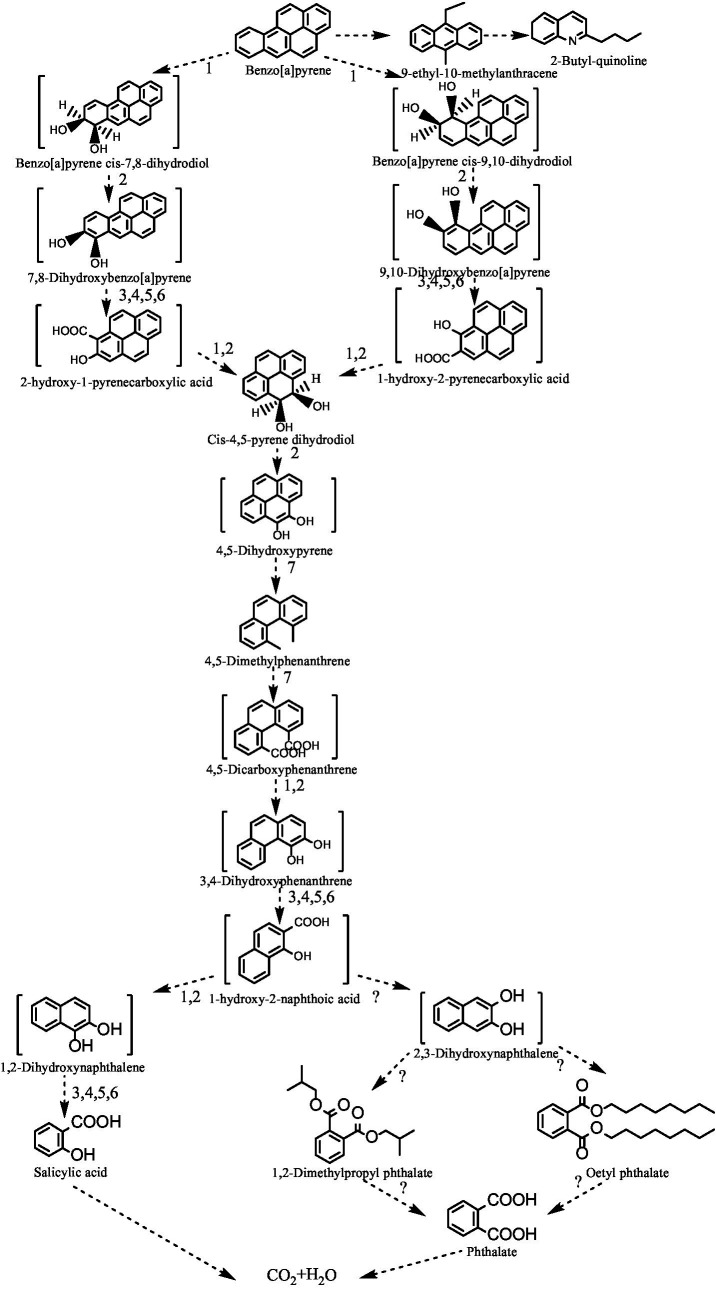
The proposed metabolic pathway of strain M1 degrading BaP. Arrows labeled with 1 represent hydroxylation steps by dioxygenases; arrows labeled with 2 identify cis-dihydrodiol dehydrogenase, activity; steps 3, 4, 5, 6 involve reactions catalyzed by an o-diphenol dioxygenase, 2-hydroxychromene-2-carboxylic acid isomerases, trans-o-hydroxybenzylidenepyruvate hydratase-aldolases, and salicylaldehyde dehydrogenases, and 7 represents activity of an o-diphenol dioxygenase. Arrows labeled ? represent steps by unknown enzyme(s).

We propose a tentative BaP catabolic pathway as shown in Figure 4, based on the observations presented here and on interpretations of previous literature data on metabolic pathways (Huang, 2015). The catabolism of BaP in M1 possibly begins with hydroxylation of the C-7/C-8 bond by a hydroxylation dioxygenase to form BAP-cis-7,8-dihydrodiol (top left of Figure 4). Alternatively, the first attack occurs at the C-9/C-10 bond to produce BaP-cis-9,10-dihydrodiol (top right of Figure 4). When formed, BaP-cis-7,8-dihydrodiol is catabolized into cis-4,5-pyrene dihydrodiol under the activity of dehydrogenase, dioxygenases, and decarboxylases. This intermediate can also be produced from BaP-cis-9,10-dihydrodiol. Cis-4,5-pyrene dihydrodiol is then sequentially converted to 4,5-dihydroxy pyrene, 4,5-dimethyl phenanthrene, 4,5-dicarboxy phenanthrene and 3,4 dihydroxy phenanthrene (central part of the figure). Then, 3,4 dihydroxy phenanthrene can enter the phenanthrene degradation pathway where it is converted to 1-hydroxy-2-naphthalic acid in a process involving ring cleavage through the action of a ring hydroxylation dioxygenase, dihydrodiol dehydrogenase, dioxygenase, hydration-aldolase and dehydrogenase. Subsequently, 1-hydroxy-2-naphthalic acid is catabolized through two pathways. One pathway (bottom left in Figure 4) involves its sequential conversion to 1,2-dihydroxy naphthalene and salicylic acid. Alternatively, in the pathway shown to the right, 1-hydroxy-2-naphthalic acid is oxidated to 2,3-dihydroxy naphthalene, which is further converted to dioctyl phthalate and 1,2-dimethyl propyl phthalate. In either case, the resulting intermediates are converted into carbon dioxide and water when they finally enter the tricarboxylic acid cycle.

## 4 Discussion

The available literature suggests that the utilization of various PAHs by different bacterial species can be quite different. For instance, *Pseudomonas* sp. strain y-8, *Sphingomonas* sp. strain H, *Sphingomonas* sp. strain B2-7, *Pseudomonas* sp. strain BZ-3 and a strain of *Achromobacter* sp. could grow with salicylic acid and catechol as carbon sources, with maximum utilization of 87.7 and 77.2% respectively, but these organisms could not use phthalic acid as a carbon source. This could be an indication of their ability to metabolize PAHs through the salicylic acid pathway only (Hou et al., 2018; Leihuan, 2008; Lin et al., 2014; Luo, 2008; Yang, 2013). In contrast, it has been shown that a strain of *Pseudomonas* sp. as well as *Pseudomonas aeruginosa* strain PA01 could use either salicylic acid or phthalic acid for growth (Gao et al., 2012; Zhang, 2010), leading to speculations that there may be two pathways of PAH degradation, one via salicylic acid and the other via phthalic acid. However, Baboshin and colleagues (Baboshin et al., 2008) found that the metabolic capacity of *Sphingomonas* sp. strain VKM B-2434 is relatively narrow, and most of the tested organic substances could not support growth of that strain. The *Bacillus* sp. strain M1 used in our study consumed salicylic acid, phthalic acid, gentisic acid, catechol, and protocatechuic acid as the sole carbon source, which provided a first indication that M1 might use both the salicylic acid and phthalic acid pathway to metabolize BaP.

Activity of the key enzymes phthalate dioxygenase, salicylate hydroxylase, gentisate dioxygenase, catechol-1,2-dioxygenase, catechol-2,3-dioxygenase and dehydroghenases was demonstrated in cell extracts grown in presence of BaP, and their enzyme activities positively correlated with the observed removal rate of BaP. These observations further confirmed that the degradation of BaP by strain M1 can be completed by the two metabolic pathways via salicylic acid and phthalic acid.

When analysing the intermediate metabolites of M1 metabolizing BaP in this study, phthalate derivatives were identified, similar to findings reported for the degradation of Pyr by *Bacillus* sp. strain ZS2 (Chen, 2014). This shows that phthalates are important intermediate products in PAH degradation. Alternatively, it may mean that these phthalate derivatives accumulate because they could not be further degraded. We identified metabolites containing 3 benzene rings such as 4,5-dimethylphenanthrene, 9-ethyl-10-methylanthracene, 2-ethylacridine and 9-methylacridine. The intermediate metabolites discovered here focused on the latter parts of the pathway of BaP metabolism. Unfortunately, we failed to detect any intermediates representing steps of the pyrene pathway, such as 4-ring- containing cis- and trans-4,5-pyrenedihydrodiol (Schneider et al., 1996) or intermediates of BaP containing 5-rings such as cis-7,8-dihydroxy-7,8-dihydrobenzo[a]-pyrene (Rentz et al., 2008). Others have detected 4,5-dimethylphenanthrene when *Pseudomonas* sp. strain JP1 or *Microbacterium* sp. strain CSW3 metabolized BaP (Liang et al., 2014; Qin et al., 2017). At present, cis-4,5-pyrene dihydrodiol and 1-hydroxy-2-naphthalic acid remain hypothetical intermediates in the pathway of *Bacillus* sp. M1 metabolizing BaP. In addition, to our knowledge the detection of 9-ethyl-10-methylanthracene and 2-ethylacridine has not been described in the metabolic pathways of BaP before, suggesting that alternative metabolic routes may exist in *Bacillus* sp. M1 metabolizing BaP, which needs to be further explored. Likewise, at least one metabolite containing two benzene rings, 2-butyl-quinoline, was detected, which has not been mentioned in other studies so far.

We furthermore report the detection of silylated esters that were most likely formed during GC/MS analysis from organic acids, such as 2,5-dihydroxyacetophenone, 2-hydroxy-5-methylbenzoic acid, salicylic acid acid and catechol. Based on the identified metabolites and key enzyme activities, combined with previous published results, the most likely biodegradation pathway of *Bacillus* sp. M1 metabolizing BaP is proposed here. The metabolic pathway of BaP has been proposed for *Pseudomonas* sp. strain JP1, *Microbacterium* sp. strain CSW3, *Cellulosimicrobium cellulans* strain CWS2 and *Stenotrophomonas maltophilia* (Chen et al., 2013; Liang et al., 2014; Qin et al., 2018; Qin et al., 2017), in which metabolites of pyrene like 1,12-dimethylbenzo[a]anthracene, 7,8,9,10-tetrahydrobenzo[a]pyrene and 5-ethylpropene were detected, but not low-molecular weight salicylate or phthalate derivatives. As a result, a metabolic chain had been speculated from BaP to anthracene or phenanthrene. Others had detected the phenanthrene metabolites such as 4,5-dimethylphenanthrene, 4-methylphenanthrene and 2,3-dimethylphenanthrene, and the naphthalene metabolites 2-methyl-1-naphthaleneacetic acid and 5,8-dihydrogen-1-naphthol (Qin et al., 2017). These different metabolites, identified for different species, suggest the presence of different metabolic pathways. Our study with *Bacillus* sp. M1 metabolizing BaP detected products similar to previous reports, as well as a number of newly discovered intermediate metabolites.

Our study has a few limitations. Due to experimental limitations related to the limited accumulation and rapid turnover of metabolites, we were unable to identify all intermediate products of BaP as it is metabolized to Pyr. Likewise, products of phenanthrene being degraded to 1-hydroxy-2-naphthoic acid were not detected. Lastly, only extracellular metabolites were investigated here. This means that the proposed pathway is not yet complete. Despite these limitations, the proposed pathway for BaP degradation provides novel insights. Further details of BaP metabolism by *Bacillus* sp. strain M1 will require future research, to identify more metabolites in order to determine the complete pathway in all of its details.

## 5 Conclusion

*Bacillus* sp. strain M1 was shown to grow on and metabolize salicylic acid, phthalic acid, gentisic acid, catechol, protocatechuic acid as the sole carbon source. When BaP is offered as the sole carbon source, it is degraded by M1 either by the salicylic acid pathway or by the phthalic acid pathway. During this degradation, 17 different intermediates were demonstratedly formed, including 4,5-dimethylphenanthrene, 9-ethyl-10-methylanthracene, phthalate derivatives and silylated catechol. In particular, phthalate derivatives accumulated prominently. All available evidence suggests that the initial position of BaP attack occurs at the C-9/C-10 or the C-7/C-8 bond. Enzymic activity results in the formation of a Pyr derivative, whose ring is opened to generate 4,5-dimethylphenanthrene. Enzyme activity of relevant enzymes was demonstrated that significantly positively correlated with the degradation rate of BaP. Ultimately, 1-hydroxy-2-naphthoic acid is formed that can be digested to phthalic acid and salicylic acid by two parallel downstream metabolic pathways. The phthalic acid pathway seems to be the more abundant of the two in *Bacillus* sp. strain M1. In addition, two new products, 9-ethyl-10-methylanthracene and 2-ethylacridine, were discovered when *Bacillus* sp. strain M1 degraded BaP. Their formation needs to be explored in depth, as they may be indicative for alternative, novel metabolic pathways.

## Data Availability

The original contributions presented in the study are included in the article/supplementary material, further inquiries can be directed to the corresponding authors.
